# Effects of polyaluminum chloride and lanthanum-modified bentonite on the growth rates of three *Cylindrospermopsis raciborskii* strains

**DOI:** 10.1371/journal.pone.0195359

**Published:** 2018-04-03

**Authors:** Fabiana Araújo, Frank van Oosterhout, Vanessa Becker, José Luiz Attayde, Miquel Lürling

**Affiliations:** 1 Programa de Pós-Graduação em Ecologia, Universidade Federal do Rio Grande do Norte, Natal, Rio Grande do Norte, Brazil; 2 Laboratório de Recursos Hídricos e Saneamento Ambiental, Departamento de Engenharia Civil, Centro de Tecnologia, Universidade Federal do Rio Grande do Norte, Natal, Rio Grande do Norte, Brazil; 3 Aquatic Ecology and Water Quality Management Group, Department of Environmental Sciences, Wageningen University, Wageningen, The Netherlands; 4 Departamento de Ecologia, Centro de Biociências, Universidade Federal do Rio Grande do Norte, Natal, Rio Grande do Norte, Brazil; 5 Department of Aquatic Ecology, Netherlands Institute of Ecology (NIOO-KNAW), Wageningen, The Netherlands; INRA, FRANCE

## Abstract

In tropical and subtropical lakes, eutrophication often leads to nuisance blooms of *Cylindrospermopsis raciborskii*. In laboratory experiments, we tested the combined effects of flocculant polyaluminum chloride (PAC) and lanthanum-modified bentonite (LMB) on the sinking and growth rates of three *C*. *raciborskii* strains. We tested the hypothesis that the combination of PAC and LMB would (1) effectively sink *C*. *raciborskii* in a test tube experiment and (2) impair *C*. *raciborskii* growth, irrespective of the biomass of the inoculum (bloom) and the strain in the growth experiment. We tested the recommended (LMB1) and a three-times higher dose of LMB (LMB3). The combined addition of PAC and LMB enhanced the sedimentation of all *C*. *raciborskii* strains. Moreover, both the PAC and LMB doses decreased the phosphate concentration. PAC and LMB1 decreased the growth rate of all strains, but the efficacy depended on the biomass and strain. The combined addition of PAC and LMB3 inhibited the growth of all strains independently of the biomass and strain. We conclude that a low dose of PAC in combination with the recommended dose of LMB decreases *C*. *raciborskii* blooms and that the efficiency of the technique depends on the biomass of the bloom. A higher dose of LMB is needed to obtain a more efficient control of *C*. *raciborskii* blooms.

## Introduction

The eutrophication of lakes and reservoirs often leads to blooms of cyanobacteria, which is considered the most important water quality problem worldwide [1,2]. Due to their toxicity, cyanobacteria blooms and their associated surface scums render water from freshwater ecosystems unfit for human use [3]. The filamentous cyanobacterium *Cylindrospermopsis raciborskii* (Woloszynska) Seenaya and Subba Raju has received great attention because of its potential toxicity and problematic high densities in eutrophic lakes and reservoirs [4–6]. The success of *C*. *raciborskii* has been attributed to its high uptake and storage capacity of phosphorus [7–10] among other factors. Many reservoirs in Brazil that are used as a water supply suffer from persistent blooms of *C*. *raciborskii* [6,11–15], which may pose a risk to human health.

Eutrophication control, hence mitigating cyanobacterial blooms, primarily focuses on phosphorus (P) control [16]. Decreasing the external P loading is a prerequisite for water quality improvement [17,18]. However, lakes often show little signs of recovery in response to decreasing the external P load, which is generally because of internal P loading from P-rich sediments[18–20]. For this reason P is one of the main causes of unsuccessful mitigation attempts [21]. Therefore, additional actions are often needed to decrease this internal P loading and accelerate lake recovery [17,22]. Internal P loading can be decreased by removing P rich sediments—dredging [23]—or by applying a P-fixative as an in-lake treatment which is often a far cheaper option than dredging [18,22]. Aluminum, calcium, and iron salts have long been applied as a P-fixative in lakes [22]. Recently, solid phase P sorbents (SPB) have gained interest [24], among which are modified claysthat is clays enriched with aluminum [25], iron [26], and lanthanum [27]. Among these SPB, anthanum-modified bentonite (LMB; Phoslock^®^) is the most widely used and tested [24]. LMB is effective in removing dissolved P from the water column and in decreasing the release of P from the sediment after it has settled on the lake bed [24]. LMB contains 5% lanthanum [28] and has a strong affinity to bind oxyanions, especially phosphate [29,30]. Thus, whole-lake application of LMB is considered a promising method to mitigate eutrophication [27,31–35].

Because LMB only targets phosphates, it does not directly affect the phosphorus present in biota. Because cyanobacteria such as *C*. *raciborskii* have a high P uptake and storage capacity [8], a bloom may prevail after the application of LMB. Lürling and van Oosterhout (2013) combined LMB with a low dose of flocculant (polyaluminium chloride, or PAC) to instantaneously achieve a durable mitigation of persistent blooms of cyanobacteria *Aphanizomenon flos-aquae* in a Dutch lake. This “flock and lock” treatment removes the total P from the water column through flocculation, using LMB as both the sinking weight and sediment-capping P fixative [36]. Several nuisance cyanobacteria contain gas vesicles that provide positive buoyancy, allowing them to accumulate at the water surface [37]. Therefore, the added sinking weight is essential, along with a low dose of flocculant, to effectively sink buoyancy-controlled cyanobacteria. The “flock and lock” method yielded good flocculation and sinking of cyanobacteria in a short-term (2 hours) laboratory experiment and in a whole-lake applications. A developing bloom of *A*. *flos-aquae* was effectively precipitated out of the water column in Lake Rauwbraken [36] and Lake De Kuil [38]. Both lakes remained devoid of cyanobacteria blooms for a long period. The “flock and lock” treatment also aims to strongly control the growth of cyanobacteria via P limitation. The prime active ingredient is LMB, which that effectively hampers the growth of the cyanobacterium *Microcystis aeruginosa* and efficiently controls a blooming concentration of *Anabaena* [35]. However, no data on the effect of the “flock and lock” treatment on the growth of cyanobacteria has been published; thus, it is unknown if the efficacy of the method is affected by the amount of cyanobacterial biomass present during application.

Although the “flock and lock” technique seems promising in removing and controlling cyanobacteria through sinking and P limitation there is no report on the efficacy of this method for controlling *C*. *raciborskii* blooms. In Brazilian reservoirs, particularly in the northeast where there are perennial blooms of *C*. *raciborskii*, a low biomass can only be reached in the rainy season [11], but mostly sites with perennial blooms contain higher chlorophyll-*a* concentrations [39] and a varying biomass throughout the season [11]. Therefore, we first tested the hypothesis that the combination of PAC and LMB would effectively sink a blooming concentration of a positive buoyant *C*. *raciborskii* to the bottom of test tubes regardless of the strain used. Hence, we tested the hypothesis that the combination of a low PAC dose and the manufacturer’s recommended LMB dose, as well as a higher dose if needed [40], would impair *C*. *raciborskii* growth, irrespective of the inoculum (bloom) biomass and the strain, in a 5 day growth experiment.

## Materials and methods

### Chemicals

The flocculant PAC (polyaluminum chloride, with the general formula Al_n_(OH)_m_Cl_3n-m_; Ekofix) was provided by Sachtleben Wasserchemie GmbH (Germany). The lanthanum-modified bentonite (LMB)—Phoslock^®^ (5% La) was supplied by Phoslock Europe GmbH (Ottersberg, Germany).

The manufacturers recommend a LMB dose of LMB (g): P (g) = 100: 1. In the growth experiment described below, LMB is applied at one (LMB1) and three (LMB3) times the recommended dose—as based on the filterable reactive phosphorus (FRP) concentrations measured at the start of each experiment.

### Strains

Three clonal non-axenic strains of the cyanobacterium *C*. *raciborskii* were used. The German *C*. *raciborskii* strain G75 was provided by Dr. Jutta Fastner (Federal Environmental Agency, Berlin, Germany). The French (PMC 124.12) strain was obtained from the Museum National d’Histoire Naturelle (Paris, France). The Brazilian strain CYRF1 was obtained from the Laboratório de Ecofisiologia e Toxicologia de Cianobactérias, Federal University of Rio de Janeiro (Rio de Janeiro, Brazil).

The strains were cultured in a slightly modified WC medium [41], in an incubator at 27°C under constant orbital shaking of 60 rpm and a photoperiod of 14:10 h light:dark. Day–night transitions were simulated through a gradual increase (or decrease) of the light intensity from complete darkness up to an approximate 130 μmol photons m^-2^ s^-1^. Stock cultures were transferred to fresh sterile medium every 3–4 weeks.

### Sinking experiment

The experiment was undertaken using a complete 2 x 2 factorial design with PAC and LMB concentrations as factors and three replicates per cell. Factor PAC had two levels: no addition (control) and the addition of 1 mg Al l^-1^. Factor LMB had two levels: no addition (control) and the addition of 0.1 g l^-1^ LMB. Aliquots of stock cultures of each *C*. *raciborskii* strain were diluted in freshly prepared WC medium. This dilution aimed at an approximate 100 μg l^-1^ chlorophyll-*a* (CHL-*a*) per strain. From each of the diluted *C*. *raciborskii* suspensions, 125 mL aliquots were distributed over 12 glass tubes. PAC was first added in the solution to achieve the final concentrations of 1 mg Al l^-1^ in treatments with PAC, and then the suspensions were mixed. This was immediately followed by adding LMB, which was done by making a slurry with 5 mL water from the tube that, was then sprayed on the top of the tube using a pipette. All tubes were incubated at room temperature (around 20°C) with no shaking for 1 day (24 h for CYRF and 20h for G75 and PMC124.12).

Because *C*. *raciborskii* is a buoyancy-controlled cyanobacteria, the accumulation of biomass (e.g., scum formation) in the top of the tubes is expected. Hence, at the end of the incubation periods, CHL-*a* concentrations and turbidity were measured in the top 10 mL and in the bottom 10 mL samples from each tube. CHL-*a* was measured with the PHYTO-PAM phytoplankton analyzer (Heinz Walz GmbH, Germany), and the turbidity was determined using a Hach 2100P turbidity meter.

### Growth experiment

A 5 day growth experiment was undertaken for each strain using a 4 x 3 factorial design where four initial biomasses were combined with three “flock and lock” treatments with three replicates per cell. The factor “initial biomass”–simulated different bloom conditions measured as the CHL-*a* concentrations (μg l^-1^) at the beginning of the experiment—this factor had levels CHL-*a*: B1 ≈ 40 μg l^-1^, B2 ≈ 80 μg l^-1^, B3 ≈ 180 μg l^-1^, and B4 ≈ 380 μg l^-1^. Samples were taken from each diluted culture to assess the FRP concentration in the medium and determine the quantity of LMB to be applied. The levels of the factor “treatment” were as follows: control (no addition), combined addition of 1 mg l^-1^ PAC and 100 g LMB: 1 g FRP (LMB1) and combined addition of 1 mg l^-1^ PAC and 300 g LMB: 1 g FRP (LMB3). Initial biomasses were achieved through the appropriate dilution of the stock cultures using the WC medium. When applying the treatments, each experimental unit first received the PAC to achieve the final concentration of 1 mg Al l^-1^. Then, after the experimental units were mixed and to improve flock formation, the pH was adjusted to 6.5 (±0.2) using hydrochloric acid (HCl 0.01N). Finally, LMB was added by making a slurry with 5 ml water from the experimental units, and the experimental units were mixed again. The experiments were undertaken in 100 ml Erlenmeyer flasks with 100 ml of the diluted cyanobacteria cultures. The incubation was done under the same conditions described for the stock cultures.

To quantify the FRP depletion, samples were collected just before, 1, 3 and 5 days after the application of PAC and LMB. Filtered samples were analyzed for their FRP concentration [42] in a segmented flow auto-analyzer (SKALAR SA40). The growth was assessed by a daily CHL-a measurement as a proxy for biomass. The growth rate μ (d^-1^) was computed by the formula μ = (ln*B*_*t*_−ln*B*_*0*_)/t, where *B*_*t*_ and *B*_*0*_ are the biomass at the end and at the start of the experiment respectively, while *t* is the duration of the experiment (5 days). The growth rate was estimated only for the control and LMB1 dose where the strains showed an exponential growth. Also, we compared the effect of tLMB3 dose in decreasing biomass with the LMB1 dose by calculating the percentage of biomass (%) decreased by treatment in relation to control by the formula % B = 100 x ((B_C_−B_T_)/B_C_), where B_C_ and B_T_ are the biomass concentration at the control and treatment respectively.

### Data analysis

To evaluate the isolated and combined effects of PAC and LMB on CHL-a concentration and turbidity in the top and bottom of the test tubes experiment, we performed two-way ANOVAs with the strain and treatment as the fixed factors. Likewise, in the growth experiment, we used a two-way ANOVA to evaluate the isolated and combined effects of the biomass and”flock and lock” treatments on FRP concentration and *C*. *raciborskii* biomass and growth rates for each strain separately. The Tukey post-hoc test was performed when the ANOVA showed significant effects of the”flock and lock” treatments in the growth experiment.

## Results

### Sinking experiment

**Fig 1:**
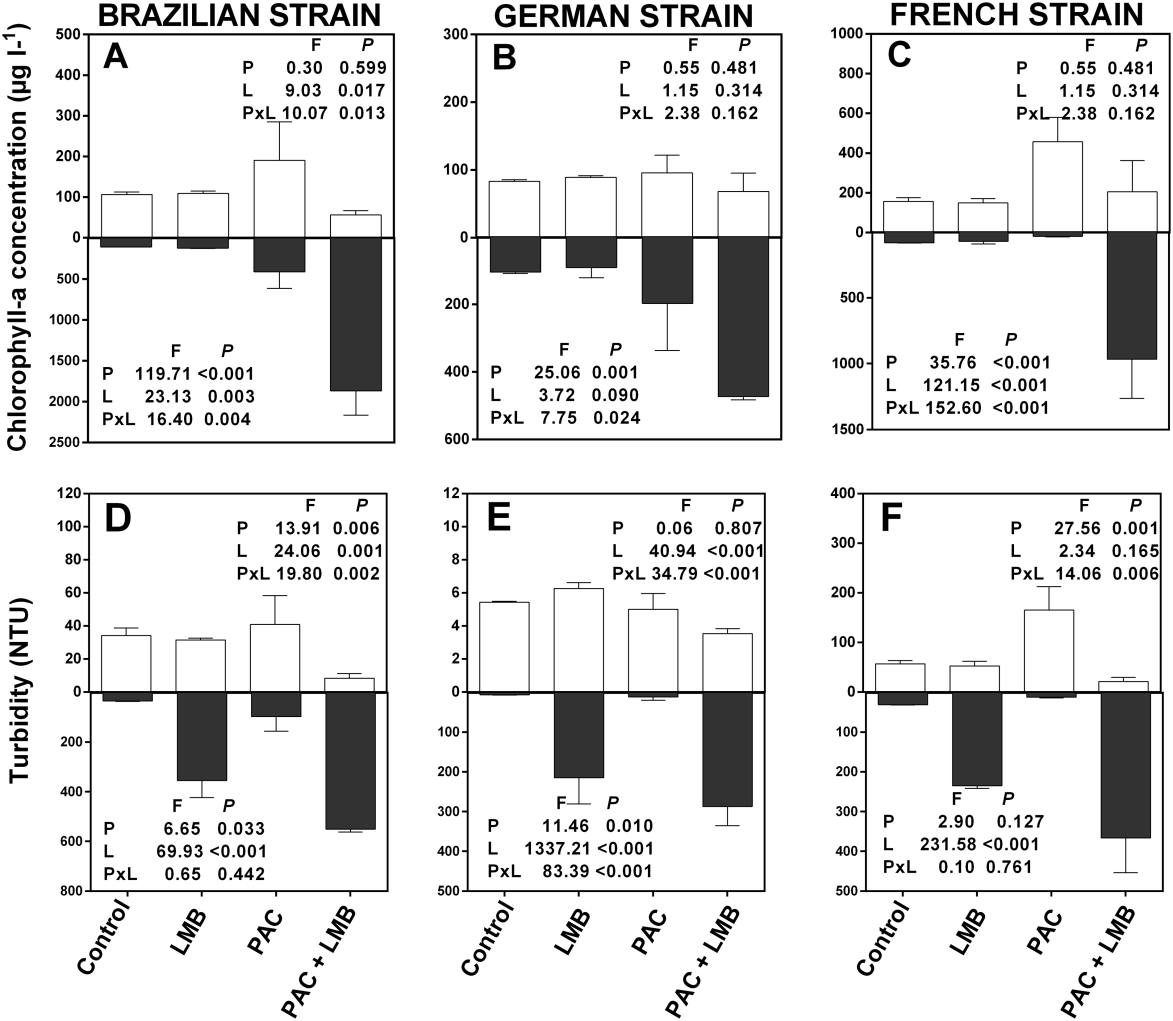
Chlorophyll-a concentrations and turbidity at the end of the incubation in the top 10 ml (white bars) and bottom 10 ml (black bars) of the experimental tubes containing 100 ml of three different *Cylindrospermopsis raciborskii* strains. Control and treatments with addition of 0.1 g l^-1^ LMB and 1 mg l^-1^PAC in isolation (LMB, PAC) or in combination (PAC+LMB) (LMB: lanthanum modified bentonite; PAC: polyaluminum chloride). F-ratios and P-values of two-way ANOVA to test for the effects of PAC (P), LMB addition (L), and their interactions (P x L). Values were considered significant assuming P ≤ 0.05.

Before applying the treatments the mean CHL-*a* concentration in the tubes was 85.3 μg l^-1^ (± 1.0) for the Brazilian strain [CYRF1], 102.7 μg l^-1^ (± 1.1) for the French strain [PMC 124.12] and 112.7 μg l^-1^ (± 0.5) for the German strain [G75], and the mean turbidity in the tubes was 31.2 NTU (± 1.6) for the Brazilian strain, 34.7 NTU (± 1.8) for the French strain and 4.9 NTU (± 0.4) for the German strain. After the incubation period the LMB treatment did not decrease the CHL-*a* concentration in the top samples (Fig 1a–1c). The PAC treatment caused a substantial increase in CHL-*a* of the top samples of the Brazilian and French strains (Fig 1a and 1b), while a minor increase was observed for the German strain (Fig 1c). When added in concert, PAC and LMB caused a decrease in the CHL-*a* concentration in the top samples and a marked increase in the CHL-a concentration of the bottom samples (Fig 1a–1c). A similar pattern was observed for turbidity. The combined addition of PAC and LMB resulted in a decreased turbidity in the top samples and an increase in turbidity in the bottom samples (Fig 1d–1f). However, the observed increase in turbidity of the bottom samples occurred when LMB was added either in isolation or in combination with PAC. The two-way ANOVA results showed a significant interaction between LMB and PAC effects in increasing CHL-*a* in the bottom samples and decreasing the turbidity in the top samples of all strains (Fig 1).

### Growth experiment

**Fig 2:**
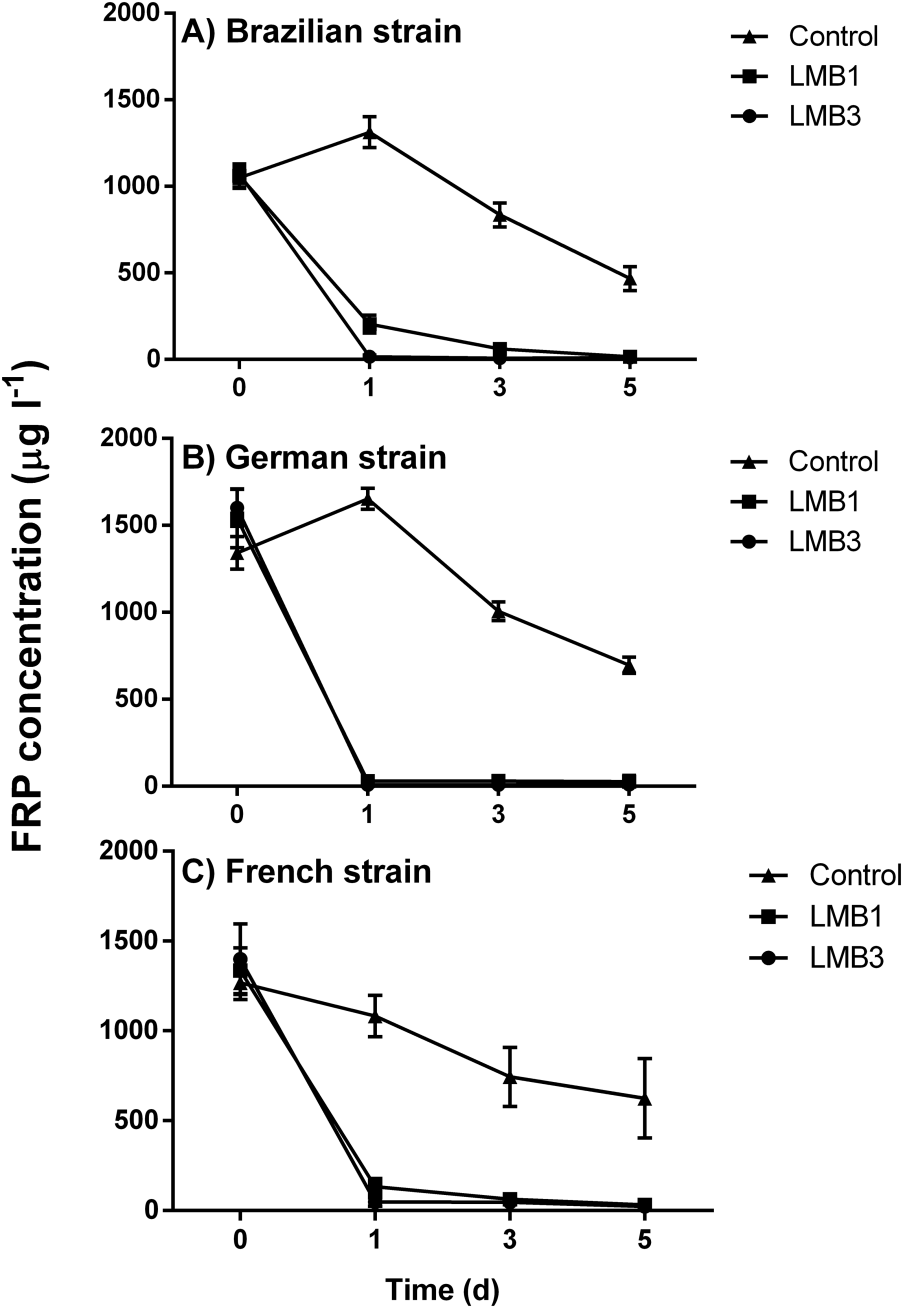
Variation of filterable reactive phosphorus concentration (FRP) during the incubation period (5 days) of experimental cultures containing 100 ml of each of the three different *Cylindrospermopsis raciborskii* strains. Treatments: Control (triangles), addition of 1 mg l^-1^ PAC + 100 g LMB: 1 g FRP (squares) and the combination of 1 mg l^-1^ PAC + 300 g LMB: 1 g FRP (circles) (LMB: lanthanum modified bentonite; PAC: polyaluminum chloride).

Because we did not observe differences in the FRP concentration with the initial biomass, the data of FRP concentration were pooled for each density (Fig 2). FRP concentrations in the controls gradually declined when *C*. *raciborskii* grew, but remained much higher than in the LMB treatments. The effect of the LMB dose is also reflected in the residual FRP concentrations determined after 1 day (Fig 2). Kruskal-Wallis One Way Analysis of Variance on Ranks (because the assumptions for a one-way ANOVA were violated) for each strain indicated that the FRP after 1 day was the highest in the controls, significantly lower in the LMB1 treatments and lowest in the LMB3 treatments (Brazilian strain *H*_2_ = 31.1; *P* < 0.001; French strain *H*_2_ = 29.3; *P* < 0.001; German strain *H*_2_ = 30.7; *P* < 0.001). In the controls FRP concentrations over the course of the experiment declined on average by 56% for the Brazilian strain, 50% for the French strain and 48% for the German strain. In the LMB1 dose these decreases were 99%, 98% and 98%, respectively, while in the LMB3 dose, FRP was on average reduced by 99%, 98% and 99% in incubations with the Brazilian, French, and German strains, respectively (Fig 2).

The growth curves revealed an exponential growth of *C*. *raciborskii* in the control and LMB1 treatments during the 5 days of the experiment (S1 Fig). In the LMB3 treatment, *C*. *raciborskii* did not show exponential growth until the end of the experiment, but in most cases declined in biomass (S1 Fig). Therefore, the growth rates were only estimated for the control and LMB1 treatments.

**Fig 3:**
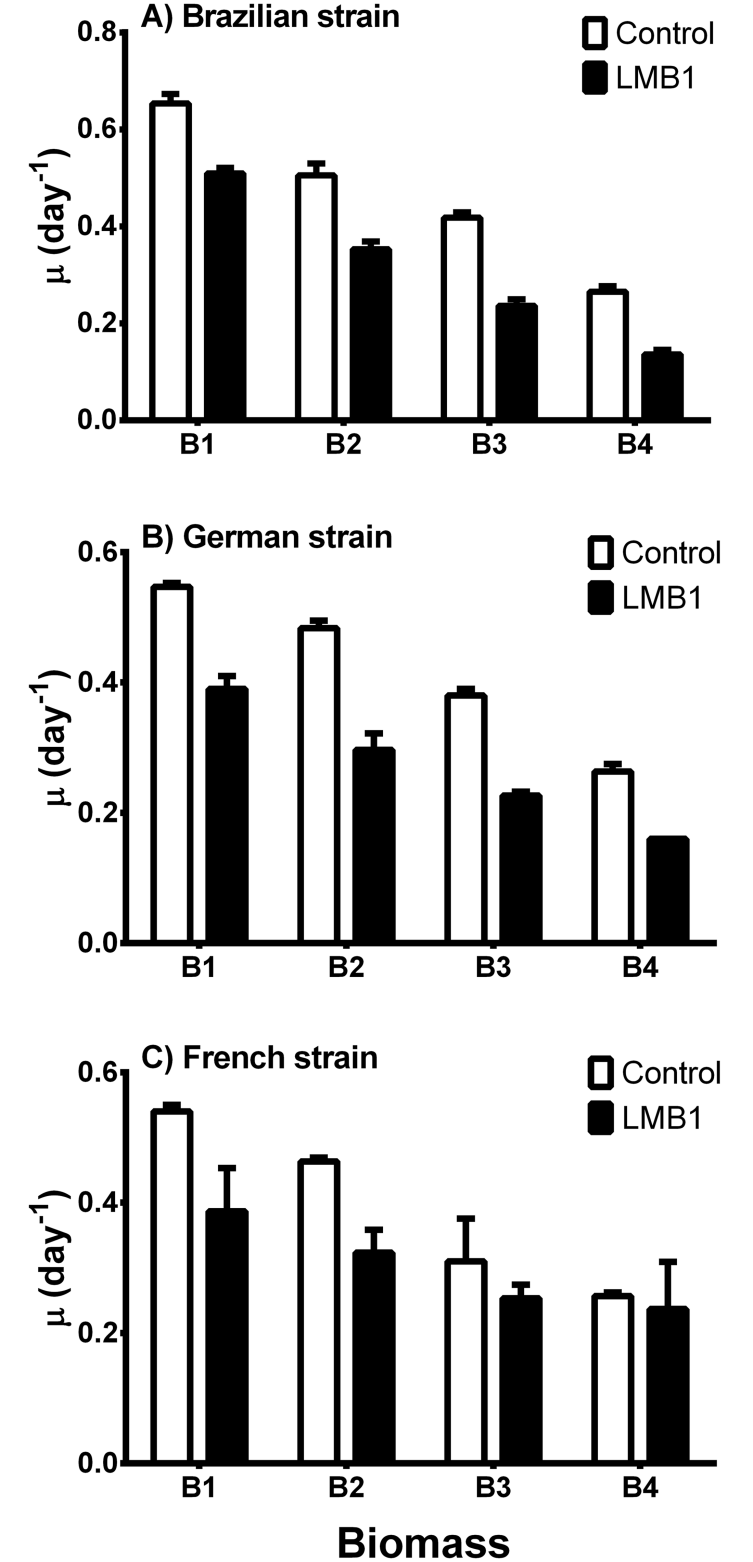
Mean (± 1SD) growth rates (μ in day^-1^) of the *Cylindrospermopsis raciborskii* strains during the incubation period (5 days) of the experimental cultures without (white bars) and with (black bars) the combined additions of 1 mg l^-1^ PAC and 100 g LMB: 1 g FRP (LMB: lanthanum modified bentonite; PAC: polyaluminum chloride). Initial biomass (chlorophyll concentrations) of the strains are Biomass 1 ≈ 40 μg l^-1^, Biomass 2 ≈ 80 μg l^-1^, Biomass 3 ≈ 180 μg l^-1^, and Biomass 4 ≈ 380 μg l^-1^.

The growth rates were higher in the control than in the treatment with the PAC + LMB addition (LMB1), decreasing with increasing the initial biomass (Fig 3). We observed a decrease of 22–29% for biomass B1, 30–39% for biomass B2 in all strains and 40–44% for biomass B3 and 40–49% for biomass B4 in the Brazilian and German strains. The two-way ANOVA results showed that the initial biomass and the PAC + LMB addition (LMB1) had significant effects on the growth rates of all strains, and that there was a significant interaction between these effects. No significant difference was found in the growth rate between the treatments in biomass 3 and 4 for the French strain.

**Fig 4:**
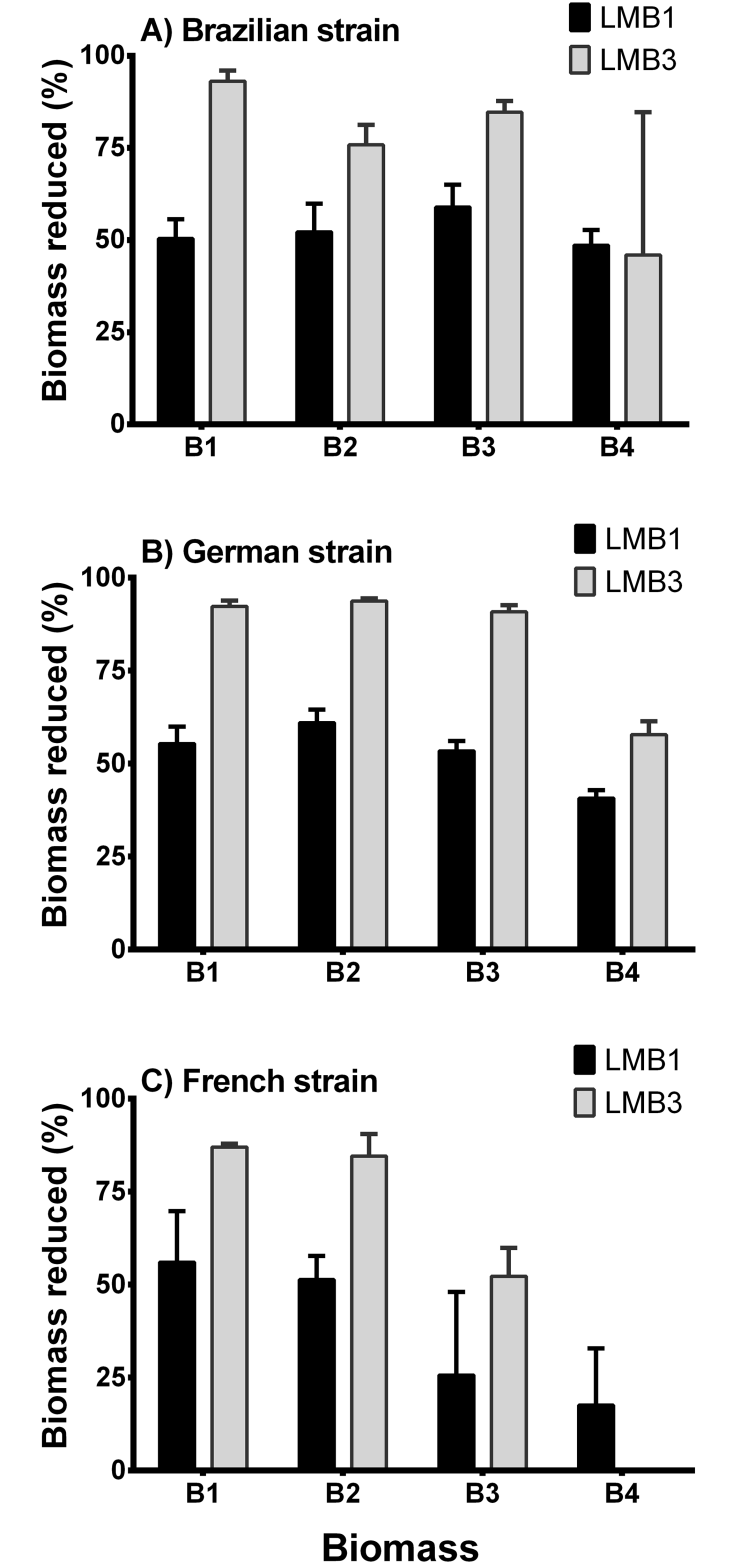
Percentage of biomass that decreased in relation to the control after the incubation period (5 days) of the experimental cultures containing 100 ml of each of the three different *Cylindrospermopsis raciborskii* strains. Treatments: addition of 1 mg l^-1^ PAC + 100g LMB: 1g FRP (black bars) and the combination of 1 mg l^-1^ PAC + 300 g LMB: 1 g FRP (gray bars) (LMB: lanthanum-modified bentonite; PAC: polyaluminum chloride); Initial biomass of the strains in chlorophyll-a concentration are Biomass 1 ≈ 40 μg l^-1^, Biomass 2 ≈ 80 μg l^-1^, Biomass 3 ≈ 180 μg l^-1^, and Biomass 4 ≈ 380 μg l^-1^.

The LMB1 dose decreased up to 61% of the biomass, while the LMB3 dose decreased up to 94% of the biomass for all strains (Fig 4). In general, the decrease of the biomass was lower with the increase in initial biomass.

## Discussion

Our results from the laboratory experiments show that the combined application of PAC and LMB could potentially be an efficient technique to effectively sink the buoyant cyanobacterium *C*. *raciborskii* out of a water column. The clay efficiency in removing cells can be enhanced by flocculant addition because of an increase in clay adhesiveness [43]. Previous studies have reported that the combination of clay and flocculant can efficiently remove marine algal cells and blooms [44–47] and freshwater cyanobacteria blooms [36,38,48] from a water column. Clay alone had been reported to effectively remove *Microcystis* cells by sinking [49–51]. We did not find the same result for *C*. *raciborskii*. LMB alone did not result in a decrease of the CHL-a concentration in the top of our experimental tubes. The increasing turbidity at the bottom of the tubes in the LMB treatment can be explained by the clay settling to the bottom, because there was no decrease in CHL-a at the top of the tubes or increase in CHL-a at the bottom of the tubes in the LMB treatment. This reveals the low aggregation efficiency of *C*. *raciborskii* filaments with LMB. Aggregation with clay may depend on the cyanobacteria used because of variability in the extracellular polysaccharide (EPS) composition, as has been found for *Microcystis* [51]. In fact, the colonies of *Microcystis* are embedded in mucilage, which is formed mainly by polysaccharide [52]. Also *Raphidiopsis brookii*, which is closely related to *C*. *raciborskii*, produces EPS [53], but we are not aware of studies showing EPS production by *C*. *raciborskii*. Conversely, we observed that the addition of PAC resulted in good flock formation as demonstrated by the increasing CHL-*a* concentrations in the top of the tubes of the sole PAC treatments, but we did not observe sedimentation of these flocks. Therefore, our results show that the addition of sinking weight in combination with a low dose of flocculant is fundamental to effectively sink the filaments of the buoyancy-controlled cyanobacterium *C*. *raciborskii*. This corroborates with other studies that also showed the sinking weight as being essential in settling positively buoyant cyanobacteria [36,38,48].

In the growth experiment, the application of PAC in combination with the recommended or a three-times higher dose of LMB equally resulted in a strong decrease of FRP concentrations by 98–99% after 5 days. We observed that even in the artificial growth medium (modified WC medium), which also has other oxyanions LMB at the recommended dose performed well. This suggests that interference by potential confounding ligands such as carbonates and EDTA [29,54,55] are of minor importance in the medium. Nonetheless, the inhibition of *C*. *raciborskii* growth was stronger for the LMB3 compared with LMB1 dose. Because cell removal efficiency by the flocculation process increases with increasing clay concentration [47,49,50], the higher flocculation in LMB3 could have contributed to the growth inhibition of *C*. *raciborskii* strains in this treatment when compared to the LMB1 treatment. In addition, the immediate and strong P depletion in the LMB3 treatments have limited further growth of the few cyanobacteria remaining in suspension. In contrast, some FRP remained available in the LMB1 treatments that could still be harvested from the medium over the first days by the cyanobacteria. Likewise, when an *Anabaena* bloom was treated with a ~100:1, 250:1 and 500:1 LMB:FRP dose, the cyanobacteria only showed decreased growth in the 100:1 dose but were inhibited and declined in the higher doses [36].

Conversely, in the LMB1 treatment the *C*. *raciborskii* strains showed an exponential growth during the 5 days of experiment, but their growth rates decreased with the initial biomass as a result of density dependent growth [52]. This is because at a higher biomass, the availability of resources per individual is lower, resulting in stronger intraspecific competition for resources. The combined addition of PAC with the recommended dose of LMB decreased the growth rate of *C*. *raciborskii* growth rate for all the three strains that were investigated. The decrease in growth rates can be explained because of P limitation caused by the binding of FRP to PAC and/or LMB. Because the addition of PAC + LMB removed more than 90% of the initial FRP concentrations, we expected growth would dramatically decrease due to P limitation. However, growth persisted but at lower rates. This may be explained by the fact that under conditions of P limitation, *C*. *raciborskii* can regulate its physiological response to this stress, decreasing its growth rate and photosynthetic activity and increasing extracellular phosphatase activity [56]. Moreover it is known that *C*. *raciborskii* is a P storage specialist and it has a rapid phosphate uptake rate [7–10]. Under ideal circumstances, *C*. *raciborskii* can uptake excess phosphorus [7] and store it. This luxury uptake allows for the growth of two or three generations before this element becomes a limiting factor [52]. Because the cultures were not submitted to P starvation before the treatment, P might have been stored by the cells before the experiment, allowing them to grow even at lower rates with the intracellular P after the combined addition of LMB + PAC. Light limitation because of increasing turbidity in the water and flocculation of filaments caused by PAC and LMB addition can also explain the decrease in the growth rate [35].

The efficacy of the method used to decrease P was not affected by the biomass of *C*. *raciborskii* that was present at the time of application or the *C*. *raciborskii* strain used. However, we found that the efficacy of this technique for decreasing *C*. *raciborskii* growth rates depends on the biomass and strain of this cyanobacteria. The effects of PAC + LMB additions in decreasing *C*. *raciborskii* growth rates increased by increasing the biomass of the Brazilian strain and, to a lesser extent, of the German strain. By contrast, PAC + LMB additions had no effect in decreasing growth at a higher biomass (B3 and B4) of the French strain. These differences in response suggest that the Brazilian strain is more sensitive to P and maybe light limitation than the other strains. Intraspecific variations in the response to resource availability has been reported for, cyanobacteria [57–59]. Physiological differences among *C*. *raciborskii* strains in response to temperature, light intensity [60] and critical requirements for phosphorus and light [61] have also been found. Also, the existence of different physiological strains or ecotypes in *C*. *raciborskii* populations has been proposed [62,63]. Therefore, the decrease of growth in response to resource limitation may depend on the ability of the strains to optimize the uptake and the critical requirements of the resources.

These findings have important implications for the management of eutrophication of lakes and reservoirs dominated by *C*. *raciborskii*. The combination of flocculant and P sorbent effectively sank the filaments and decreased the FRP and growth rates of the *C*. *raciborskii* strains tested. These findings also suggest that the decrease in the growth rate depends on the cyanobacteria biomass and strain. Therefore we suggest that the flocculation and sedimentation associated with the reduction of internal P-loading can be a good management strategy to control *C*. *raciborskii* blooms. However, further work is required to evaluate this technique in ecosystem scale experiments and its potential impacts on the diversity and functioning of freshwater ecosystems. It is also important to point out that this technique represents a remedial and rapid measure to mitigate eutrophication effects and should be associated with the reduction of external P loading.

## Supporting information

S1 Fig Growth curves during the incubation period (5 days) of the experimental cultures containing 100 ml of each of the three different *Cylindrospermopsis raciborskii* strains without the addition of PAC and LMB (F&L 0) and with the combined additions of 1 mg l^-1^ PAC and 100 g LMB: 1 g FRP (F&L 100) (LMB: lanthanum modified bentonite; PAC: polyaluminum chloride). The initial biomass (chlorophyll concentrations) of the strains are Biomass 1 ≈ 40 μg l^-1^, Biomass 2 ≈ 80 μg l^-1^, Biomass 3 ≈ 180 μg l^-1^, and Biomass 4 ≈ 380 μg l^-1^.
(TIF)

S1 Table Variation of chlorophyll a concentrations during the incubation period (5 days) of the experimental cultures containing 100 ml of each of the three different Cylindrospermopsis raciborskii strains without the addition of PAC and LMB (F&L 0) and with the combined additions of 1 mg l^-1^ PAC and 100 g LMB: 1 g FRP (F&L 100) (LMB: lanthanum modified bentonite; PAC: polyaluminum chloride). The initial biomass (chlorophyll concentrations) of the strains are Biomass 1 ≈ 40 μg l^-1^, Biomass 2 ≈ 80 μg l^-1^, Biomass 3 ≈ 180 μg l^-1^, and Biomass 4 ≈ 380 μg l^-1^.
(XLS)
